# A role for nucleolin in functional improvement in stroke

**DOI:** 10.1016/j.isci.2026.116134

**Published:** 2026-06-01

**Authors:** Samuel P. Bridges, Mary Hovanesyan, Srbui Azarapetian, Ofri Abraham, Ida Rishal, Mike Fainzilber, S. Thomas Carmichael

**Affiliations:** 1 Department of Neurology, David Geffen School of Medicine at University of California, Los Angeles, Los Angeles, CA 90095, USA; 2 Departments of Biomolecular Sciences and Molecular Neuroscience, Weizmann Institute of Science, Rehovot 7610001, Israel

**Keywords:** molecular biology, neuroscience, cell biology

## Abstract

Stroke remains a global health concern and a leading cause of adult-onset disability due to limited intrinsic repair capability. Here, we identify nucleolin as a targetable regulator of the repair programs activated following ischemic injury. Through neuron-specific subcellular targeting of axonal nucleolin in the post-stroke recovery period, we see enhanced axonal sprouting and accelerated functional recovery following motor cortex stroke. These findings demonstrate that interference in localized nucleolin functions following cortical stroke is a viable strategy to enhance functional recovery.

## Highlights

• Nucleolin expression is increased for an extended period of time following stroke
• Neuron-specific nucleolin targeting boosts axonal sprouting and motor recovery
• Nucleolin is a viable therapeutic target for stroke recovery

## Graphical abstract

**Figure:**
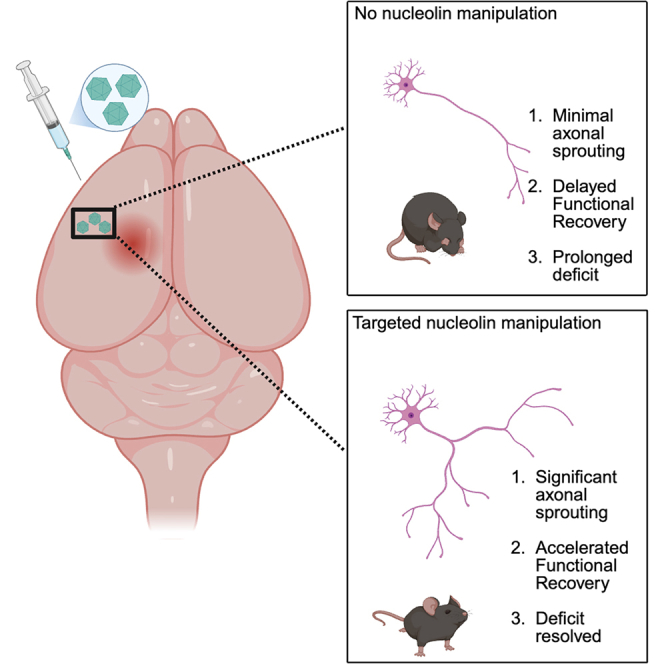

## Introduction

Stroke remains a leading cause of long-term disability worldwide, with ischemic stroke accounting for the vast majority of cases.^1^ While acute interventions such as thrombolysis and mechanical thrombectomy have significantly improved outcomes for a subset of patients, these approaches are time-sensitive and limited to the hyperacute phase.^2^^,^^3^^,^^4^ Existing therapies that are initiated beyond 24 h after symptom onset involve physical therapies with limited effectiveness. As a result, most stroke survivors experience chronic neurological deficits for which no approved regenerative therapies currently exist. The long-term impairments that follow stroke, encompassing motor, sensory, and cognitive deficits, stem not only from the acute loss of neuronal tissue but also from the failure of endogenous mechanisms to support neural repair and circuit reorganization in the injured brain.^5^^,^^6^^,^^7^^,^^8^^,^^9^^,^^10^^,^^11^

Emerging work has identified several molecular pathways that modulate plasticity and axonal regeneration following CNS injury.^11^^,^^12^ Among these, nucleolin, a multifunctional RNA- and DNA-binding protein, has gained attention for its regulatory roles in RNA metabolism, chromatin structure, and cellular stress responses. Although nucleolin is broadly expressed and best known for its nuclear functions,^13^^,^^14^ it is also present in the cytoplasm and axons of neurons,^15^^,^^16^ where it plays critical roles in the transport and localization of select mRNA transcripts.

At homeostasis, and following neuronal injury, nucleolin has been shown to bind and regulate the localization and translation of key growth-associated mRNAs, including Importin β1,^15^ mTOR,^17^ VEGF,^18^ TGF-β1,^18^ and 14-3-3ζ,^19^ all of which are implicated in axonal regeneration or retrograde injury signaling.^11^^,^^15^^,^^16^^,^^20^^,^^21^ Notably, nucleolin facilitates the transport of these transcripts from the soma into distal axons, where localized translation supports cytoskeletal remodeling, membrane dynamics, and regenerative outgrowth.^22^ However, in the case of Importin β1 and co-transported mRNAs, shifts in local translation between axon and soma can lead to changes in regenerative outgrowth rates.^15^ In peripheral nerve injury models, sequestration of nucleolin away from axons and restriction to the soma enhances regenerative capacity, likely due to altering the axonal mRNA landscape and shifting translational foci from axon to soma.^15^^,^^16^^,^^23^ Notably, nucleolin is one of the most up-regulated transcripts in spontaneously sprouting axons following stroke in aged animals,^12^ where endogenous recovery is low. Because of the dependence of nerve regeneration in the PNS on axonal levels of nucleolin,^15^^,^^22^ this general induction of neuronal nucleolin by stroke in the aged brain may indicate a potential role in limiting recovery following ischemic damage.

Given the parallels between peripheral and central injury responses, including early stress signaling and regenerative transcript mobilization^24^^,^^25^, it is plausible that nucleolin exerts a similar suppressive influence on the formation of new axonal connections and repair mechanisms following stroke as in the PNS.^26^ However, its role following ischemic brain injury remains largely unexplored. Here, we investigate nucleolin as a potential modulator of neural repair in the injured CNS. We compare nucleolin depletion through a partial knockout model to its somatic sequestration using a virus-delivered localization-perturbing domain^16^ and evaluate axonal sprouting and functional outcome measures following focal cortical stroke in male mice. We hypothesize that nucleolin upregulation inhibits post-stroke recovery and find that preventing its subcellular trafficking results in a robust increase in axonal sprouting and accelerated recovery. Our findings position nucleolin as a compelling therapeutic target for promoting recovery in the chronically injured brain.

## Results

### Nucleolin expression is induced following stroke

**Figure 1:**
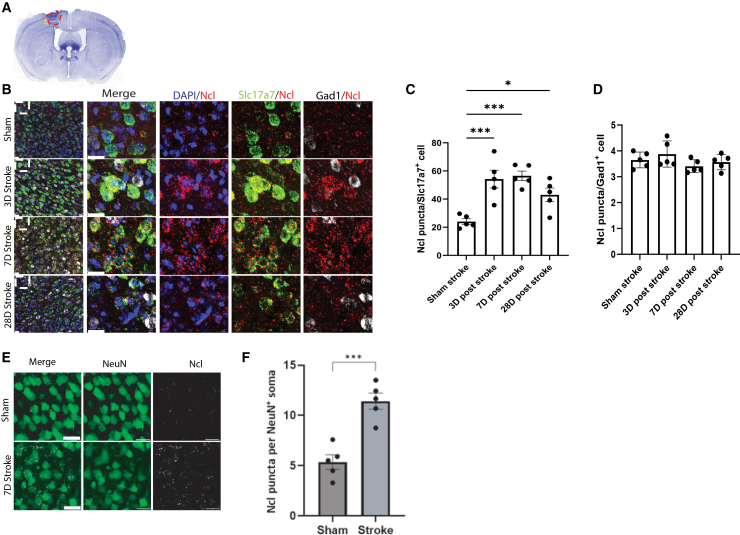
Ischemic cortical stroke increases nucleolin expression in excitatory neurons (A) Representative coronal section stained with DAPI with stroke (red dashed line) and area of interest (yellow box). (B) Representative *in situ* hybridization images of DAPI (nuclear counterstain), Slc17a7 (excitatory cortical neurons), Gad1 (inhibitory neurons), and nucleolin at time points across acute to chronic stroke. The dashed box indicates the region shown at higher resolution and magnification in accompanying panels. Scale bars, 20 μm, *n* = 5 animals per time point. (C) Quantification of nucleolin mRNA expression in excitatory cortical neurons post-stroke. Stroke induces both early and chronic expression of nucleolin in Slc17a7^+^ cells. 1-way ANOVA with post-hoc Tukey correction. Data are represented as mean ± SEM with data points representing individual animals. ∗ p = 0.039, ∗∗∗ p = 0.003 (3D post stroke), p = 0.004 (7D post stroke). (D) Quantification of nucleolin mRNA expression in inhibitory cortical neurons post-stroke. No significant change in nucleolin expression was detected in Gad1^+^ cells. Data are represented as mean ± SEM with data points representing individual animals. (E) Representative images of nucleolin protein expression in sham stroke and 7 days post-stroke mice. Scale bars, 10 μm, *n* = 5 animals per group. (F) Quantification of nucleolin protein expression post-stroke. Stroke significantly increases nucleolin expression 7 days post-stroke (*p* = 0.0005 via *t* test). Data are represented as mean ± SEM with data points representing individual animals.

A previous transcriptomic study identified nucleolin as one of the most up-regulated genes in neurons with post-stroke sprouting axons, with a 2.5--fold increase in nucleolin transcript levels in aged animals.^12^ We first validated this by performing *in situ* hybridization following the photothrombotic model of focal cortical stroke at biologically relevant time points: 3-day post-stroke, representing acute stroke, 7-day post-stroke, representing acute-on-chronic stroke and the onset of pro-growth programs as described previously,^12^ and 28-day post-stroke, representing chronic stroke and the brain’s return to a homeostatic gene program.^12^ Nucleolin mRNA expression was evaluated in Slc17a7^+^ cells, representing excitatory cortical neurons, and Gad1^+^ cells, representing cortical inhibitory neurons. When compared to no-stroke controls, stroke significantly induced nucleolin expression in stroke-adjacent Slc17a7^+^ neurons, initially at 3-day post-stroke (*p* = 0.003), peaking at 7-day post-stroke (*p* = 0.004), and remaining elevated 28-day post-stroke (*p* = 0.039) (Figures 1A–1C). This population of excitatory neurons contributes significantly to axonal sprouting following stroke. Nucleolin expression was unchanged in Gad1^+^ inhibitory cortical neurons in the same peri-infarct region (Figure 1D). There was also a 2.5-fold increase in nucleolin protein expression 7 days following stroke, the time point identified as having the greatest induction of nucleolin mRNA expression (Figures 1E and 1F *p* = 0.0005). Taken together, these results validate previous studies showing an increase in nucleolin expression following stroke.

### Partial knockout of nucleolin has a detrimental effect on post-stroke axonal sprouting

**Figure 2:**
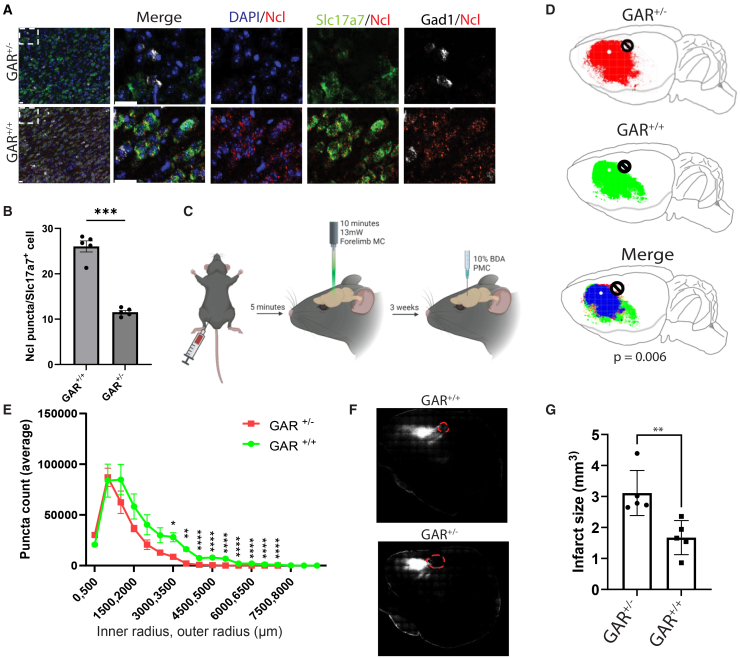
Heterozygous deletion of the nucleolin GAR domain is detrimental to post-stroke axonal sprouting (A) Representative *in situ* hybridization images show reduced nucleolin expression in GAR^+/-^ mice. The dashed box indicates the region shown at higher resolution and magnification in accompanying panels. Scale bars, 20 μm, *n* = 5 animals per group. (B) Quantification of nucleolin mRNA in GAR wild type (GAR^+/+^) and heterozygous deletion (GAR^+/-^) mice. GAR^+/-^ mice show a significant reduction in nucleolin mRNA puncta in excitatory cortical neurons (*p* = 0.004 via *t* test). Data are represented as mean ± SEM with data points representing individual animals. (C) Schematic shows the general surgery procedure. Generated via BioRender. (D) Quantitative cortical mapping of peri-infarct axonal sprouting in GAR heterozygous deletion mice compared to wild type littermates. Injection site noted via white circle. Stroke site indicated via black symbol. Heterozygous deletion of the nucleolin GAR domain significantly decreases peri-infarct axonal sprouting when compared to wild-type littermate controls (*p* = 0.006 via Hotelling’s T2 test). (E) Axonal puncta distribution within concentric rings centered at the injection origin (0,0). Heterozygous deletion of the nucleolin GAR domain significantly reduces axon detection at distances greater than 3 mm from the injection site (∗*p* = 0.0329, ∗∗*p* = 0.00286, and ∗∗∗∗*p* < 0.0001, general linear mixed model with post-hoc Tukey’s correction). Data are represented as mean ± SEM. (F) Representative cortical flat-map images of wild-type and heterozygous GAR deletion mice 1 month following cortical stroke. White circle indicates the tracer injection site, and the red dashed circle indicates the outer bounds of infarct. (G) Heterozygous deletion of the nucleolin GAR domain results in an approximately 2-fold increase in infarct size one month post stroke (*p* = 0.0078 via *t* test). *n* = 5 animals per group for all measurements. Data are represented as mean ± SEM with data points representing individual animals.

To evaluate a role for nucleolin in post-stroke axonal sprouting, photothrombotic stroke was performed on heterozygous nucleolin glycine arginine-rich (GAR) domain deletion mice^16^ (GAR^+/-^) and littermate wild-type controls (GAR^+/+^). This deletion reduces active transport of nucleolin into the axon, thus impacting the nucleolin signaling pathway as described extensively in previous work.^14^^,^^15^^,^^16^ Homozygous GAR domain deletions (GAR^-/-^) are embryonically lethal.^16^ Heterozygous deletion of the GAR domain results in an approximate 50% reduction in nucleolin mRNA in Slc17a7^+^ cells (Figures 2A and 2B *p* = 0.004 via *t* test), consistent with previous reports using this mutant mouse.^16^ Axonal sprouting was evaluated in flattened sections using the axonal tracer biotinylated dextran amine (BDA), injected anterior to the stroke one month after injury (Figure 2C). This is a site of axonal sprouting and recovery circuits in this stroke model.^6^^,^^12^^,^^27^^,^^28^ Surprisingly, GAR^+/-^ mice showed a significant decrease in axonal sprouting compared to control mice (Figure 2D, *p* = 0.006, Hotelling’s T-squared test). Quantification of axonal density, measured by concentric circles centered at the injection origin site, showed that heterozygous knockdown of nucleolin resulted in significantly lower axon density at distances greater than 3mm from the injection site (Figure 2E). Moreover, evaluation of stroke size showed an approximately 2-fold increase in stroke volume in GAR^+/-^ mice (Figures 2F and 2G, *p* = 0.0078, unpaired *t* test). This marked increase in infarct size 28 days following ischemic injury, at a time point where pro-axonal sprouting growth programs have largely terminated,^12^ indicates a persistent growth inhibitory environment. These results suggest that nucleolin has a neuroprotective effect limiting the early expansion of stroke damage, and this effect is lost in the heterozygous mutant model. The larger stroke size in the GAR^+/-^ mice likely accounts for the reduced labeling of axons adjacent to the stroke. The finding of a neuroprotective role for nucleolin early after stroke indicates that testing for its role in later recovery processes requires strict spatiotemporal control of functional targeting.

### Virally mediated somatic sequestration of nucleolin enhances post-stroke axonal sprouting

**Figure 3:**
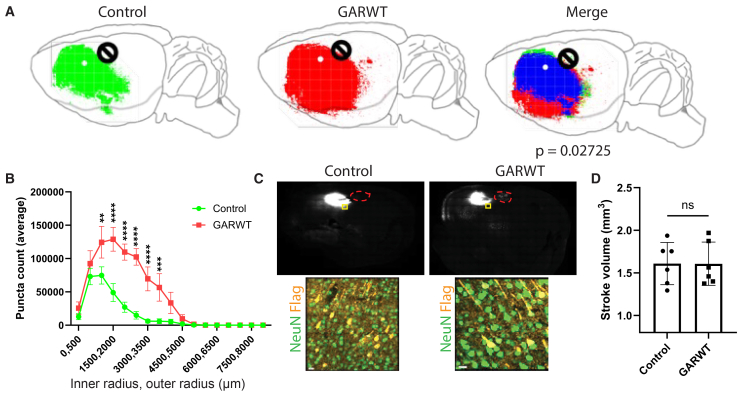
GAR-induced somatic sequestration of nucleolin enhances post-stroke axonal sprouting (A) Quantitative cortical mapping of post-stroke axonal sprouting in flattened mouse cortex ipsilateral to the stroke. Exogenous GAR-induced somatic sequestration of nucleolin, injection site noted by the white circle, results in a significant increase in axonal sprouting to the adjacent somatosensory cortex compared to Dendra2 control virus (*p* = 0.02727 via Hotelling’s T2 test). Stroke indicated via a black symbol. (B) Axonal puncta distribution within concentric rings centered at the injection site (0,0). Somatic sequestration of nucleolin significantly increases axon detection locally and distant from the injection site (∗∗*p* = 0.00234, ∗∗∗*p* = 0.000486, and ∗∗∗∗*p* < 0.0001, general linear mixed model with post-hoc Tukey’s correction). Data are represented as mean ± SEM. (C) Representative flatmap images of mice injected with control and GARWT viruses. The white circle indicates the virus injection site, and the red dashed circle indicates the border of the infarct. Yellow square indicates zoomed in image, showing colocalization of Flag and NeuN in virally transduced cells. Scale bars, 20 μm, n = 5–7 animals per group. (D) Quantification of infarct size. Somatic sequestration of nucleolin does not impact infarct size (*p* = 0.6989 via *t* test). *n* = 7 animals per group for all measurements. Data are represented as mean ± SEM with data points representing individual animals.

Given the detrimental effect of the constitutive heterozygous GAR deletion on axonal sprouting and infarct size, we next sought to target nucleolin with both cell-type and subcellular localization specificity. Within the nucleolin protein, the GAR domain controls subcellular localization by interactions with kinesin motors and membranes.^16^ Reagents targeting the GAR domain sequester nucleolin to the soma in neurons.^15^^,^^16^ We opted to express the GAR domain alone via AAV transduction to perturb axonal transport of nucleolin, leveraging tools previously shown to disrupt the subcellular localization of nucleolin.^16^ We modified existing reagents^16^ by adding a Cre recombinase and a P2A self-cleavage sequence upstream of either the GARWT-Dendra2 sequence, or Dendra2 alone, to generate a bicistronic virus and leverage Cre-mediated expression of a cell fill label. While we were unable to validate nucleolin sequestration in the brain *in vivo*, due to the technical challenges in determining the subcellular localization of proteins in intact brain tissue, the well-characterized effects of these tools in peripheral neurons^16^ motivated us to test their effects in the intact post-stroke brain. Adult male mice underwent photothrombotic stroke targeting the forelimb motor cortex and were injected in the premotor cortex with either Hsyn-Cre-P2A-dendra2GARWT (GAR overexpression virus, GARWT) or Hsyn-Cre-P2A-dendra2 (control). The HSyn promoter ensures neuron-specific expression. These were mixed with pAAV-CAG-Flex-Ruby2sm-Flag for cell labeling and axon fill of terminals. One month following stroke, a time window suitable for axonal sprouting and functional recovery,^6^^,^^12^^,^^27^^,^^28^ sprouting was assessed through the quantitative mapping of cortical connections, as described previously.^6^^,^^12^^,^^29^ Mice injected with GARWT showed a significant enhancement in axonal sprouting compared to those injected with Control (Figure 3A, *p* = 0.02725 via Hotelling’s T-squared test), with new connections preferentially mapping toward the somatosensory and insular cortices, while axonal sprouting in the control virus-treated mice followed sprouting patterns seen previously in mice that received stroke alone.^6^^,^^28^ Axon density, measured as described earlier, was significantly higher in the GARWT-injected mice, with the greatest increase in density detected within 4 mm of the injection site (Figure 3B). No significant difference in stroke size was seen (Figures 3C and 3D). These data show that neuron-specific targeting of axon-localizing mechanisms of nucleolin significantly enhances post-stroke axonal sprouting, a primary outcome measure associated with improved functional recovery following ischemic damage.

### Somatic nucleolin sequestration accelerates post-stroke functional recovery

**Figure 4:**
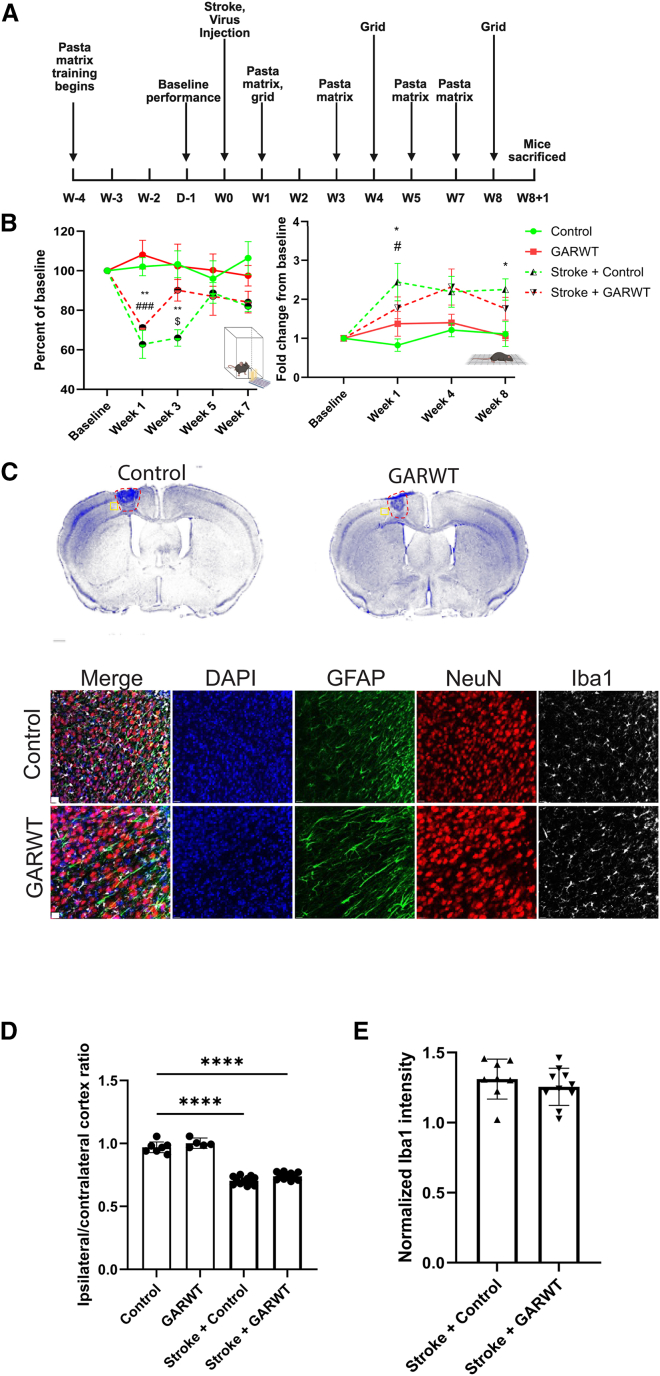
Nucleolin perturbation enhances post-stroke functional recovery (A) Diagram of grid-walking and pasta matrix tasks, and experimental timeline for task training and assessment post-stroke. (B) Somatic sequestration of nucleolin accelerates recovery in the pasta matrix fine motor control and grid-walking gross motor control tasks. Mice were evaluated for fine motor dexterity as defined by the percentage of baseline pieces of pasta broken. One-week post-stroke, both GARWT + stroke and Control + stroke mice showed a significant deficit in the number of pieces of pasta broken compared to Control alone (∗Control vs. Stroke + Control *p* = 0.0082, # Control vs. Stroke + GARWT *p* = 0.0003 via 2-way ANOVA). Three weeks post-stroke, Control + stroke mice continued to show a deficit, whereas GARWT + stroke mice had recovered to a degree with no significant difference from both control groups (∗ Control vs. Control + stroke *p* = 0.0021, Control vs. GARWT + stroke *p* = 0.4568, $ Control + stroke vs. GARWT + stroke *p* = 0.0280 via 2-way ANOVA). By five weeks post-stroke, both groups showed no significant difference from the control groups. N = 8–10 per group. Mice were evaluated for gross motor control as defined by the fold change of baseline percentage right foot faults. One-week post-stroke, Control + stroke and GARWT + stroke mice showed a significant increase in the relative number of foot faults involving the affected limb (∗Control vs. Control + stroke *p* = 0.0180, # Control vs. GARWT + stroke *p* = 0.0202 via 2-way ANOVA). At the endpoint of the experiment, 8 weeks post-stroke, Control + stroke mice showed a continued fold change increase in percent foot faults, whereas GARWT + stroke mice were not significantly different from Control alone (∗Control vs. Control + stroke *p* = 0.0323, Control vs. GARWT + stroke *p* = 0.3322). *n* = 12–15 per group. Data are represented as mean ± SEM. (C) Representative DAPI-stained cortical sections of stroke mice treated with control or GARWT virus. Yellow box outlines the peri-infarct region imaged for tissue-level outcomes, red dashed circle indicates infarct bounds. Scale bars, 20 μm. (D) Somatic sequestration of nucleolin has no effect on infarct size or tissue loss. *n* = 8 per group. *p* < 0.0001, control vs. control + stroke and control vs. GARWT + stroke via 2-way ANOVA with Tukey’s post-hoc correction. Data are represented as mean ± SEM with data points representing individual animals. (E) Somatic sequestration of nucleolin does not have an effect on Iba1 intensity following cortical stroke. *n* = 8 per group. Data are represented as mean ± SEM with data points representing individual animals.

The finding that targeting nucleolin localization enhances post-stroke axonal sprouting motivated us to test for corresponding improvements in motor function after stroke. To assess this, mice were subjected to focal cortical stroke, or sham stroke, where laser illumination occurred in the absence of Rose Bengal injection, and GAR domain or dendra2-only constructs were expressed by viral transduction as described above. Mice were then evaluated for gross and fine motor control through two validated behavioral paradigms, the gridwalking^30^ and pasta matrix^31^ tasks (Figure 4A). In the pasta matrix task, mice that received a stroke showed a significant deficit 1 week post-stroke (Figure 4B), but at 3 weeks post-stroke, mice with a GAR domain-expressing virus showed improved recovery, with no significant difference from sham-stroke mice (Figure 4B). In the gridwalking task, all mice that received stroke showed a deficit in the fold change of foot faults 1-week post-stroke compared to sham-stroke controls (Figure 4B). 8-week post-stroke, mice that received Dendra2-only injection showed a lasting deficit, but mice treated with GAR domain virus did not have a statistically significant performance compared to baseline (Figure 4B). Nuclear sequestration of nucleolin had no significant effect on infarct size (Figures 4C and 4D), as both dendra2-only + stroke and GAR domain + stroke mice had similar degrees of cortical tissue loss. IBA1, a marker for microglia and macrophages, was used to determine if there was a prolonged inflammatory environment as the peri-infarct returned to homeostasis. No change in normalized IBA1 signal intensity was seen between the two stroke groups (Figures 4C–4E). Overall, the functional output measures and chronic tissue evaluations show that a reagent that targets nucleolin localization accelerates functional recovery without prominent effects on tissue loss in the context of stroke.

## Discussion

Stroke is a leading cause of adult-onset disability, due in large part to the brain’s inability to fully regenerate following injury. Paradoxically, naturally occurring events following injury restrict the full degree of recovery that surviving neurons are capable of, with the activation of inhibitors of axonal sprouting and recovery after stroke, such as Nogo,^32^^,^^33^^,^^34^ EphrinA5,^6^ LINGO^35^^,^^36^ and MAG.^33^^,^^37^ Enhancement of axonal growth may ameliorate such effects, and modifiers of importin β1 and nucleolin trafficking have such effects in peripheral neurons.^15^^,^^16^^,^^23^ Nucleolin has previously been found to act as a “molecular brake” on axonal elongation, and knockdown or inhibition of its extra-somatic (axonal) transport in the peripheral nervous system has been found to accelerate nerve repair through the inhibition of a “stop” cue from an extending neurite.^13^^,^^14^^,^^15^^,^^16^^,^^19^ In this work, we used virally mediated the expression of the GAR domain to target endogenous nucleolin transport mechanisms in the context of stroke. This approach enabled the interrogation of the function and impact of nucleolin on post-stroke functional recovery.

We found that nucleolin GAR domain expression in cortical neurons significantly increased their post-stroke axonal sprouting. Despite the challenge in validating nucleolin sequestration in the intact brain, the positive effects seen only in those mice injected with virus driving the expression of the GAR binding domain is strong evidence of nucleolin sequestration, given the parallels seen with peripheral nervous system sequestration of nucleolin using the same approach.^16^ The axonal sprouting we observed is similar in magnitude and direction to that seen following Ephrin-A5 blockage,^6^ and CREB induction,^38^ where newly formed axonal connections are directed toward the somatosensory cortex, but is distinct from the patterns seen following the stroke-induced growth factor GDF10,^27^ with preferential axonal sprouting toward the premotor and prefrontal cortices. This could reveal distinct mechanisms of post-stroke axonal sprouting that, in the future, may be leveraged together to further enhance functional recovery. As increased axonal sprouting is causally linked to improved functional recovery, we next evaluated functional recovery through the assessment of motor activity following stroke and found that GAR expression accelerated initial motor recovery compared to mice injected with a control virus.

These results are encouraging, but given the adverse effects seen in the heterozygous GAR deletion mouse, caution is advisable when considering the timing and duration of treatments targeting nucleolin. The role of nucleolin in non-neuronal cells of the central nervous system is largely unknown. As nucleolin is widely induced in the peri-infarct cortex post-stroke, and this induction is not limited to neurons, nucleolin may play an initial role in neuroprotection post-stroke, and shift to a more growth-regulating role as the brain transitions from injury response to recovery. With this in mind, nucleolin may be an ideal target for small-molecule or other pharmacological therapy, as temporally precise inhibition of its function may allow continued operation of the pro-growth programs seen in the subacute phase of stroke, without detrimental impacts on either the acute injury response or long-term recovery. Nucleolin-targeting aptamers, such as AS1411, have been developed and evaluated in clinical trials in the cancer field, showing good safety profiles but varying therapeutic efficacy.^39^^,^^40^ Application of such an aptamer or a similarly targeted molecule after the initial insult and during the activation of endogenous growth programs could potentiate naturally occurring repair processes in a temporally precise manner. Currently available aptamers do not penetrate the blood-brain barrier, limiting their CNS applicability at present. Systemic use of such an aptamer or a targeted small molecule, once modified to cross the blood-brain barrier, could be a viable pharmaceutical strategy for future investigation.

To summarize, nucleolin is one of the most up-regulated genes in neurons actively involved in axonal sprouting post-stroke.^12^ Given the known function of nucleolin in regulating axonal elongation, overexpression or misexpression may reduce growth. Such effects could play a role in the limited recovery seen following CNS injury by reducing endogenous axonal sprouting. Our study demonstrates that targeting nucleolin localization may be a viable strategy for post-stroke manipulation.

### Limitations of the study

While these studies identify nucleolin localization as a potential therapeutic target for post-stroke functional recovery, several caveats should be considered. The detrimental effects observed in the heterozygous constitutive nucleolin knockout model underscore the limited understanding of nucleolin’s broader roles across cell types and in the early or acute cell death phases of stroke. This highlights the importance of temporal control when inhibiting nucleolin function, as premature inhibition may disrupt an initial neuroprotective effect. Additionally, although virus-mediated sequestration of nucleolin to the cell body is well validated *in vitro*, we were unable to clearly demonstrate the sequestration of nucleolin to the cell body in the intact mouse brain following the application of the same approach *in vivo*. This is because of the dense network of axonal and dendritic projections in the brain, prohibiting subcellular determination of nucleolin. While this approach of viral retraction of nucleolin from axonal localization, validated *in vitro*, was associated with enhanced axonal sprouting and improved functional recovery, it remains possible that these benefits arise from mechanisms other than restricting axonal nucleolin localization. Despite these limitations, this study is the first to establish a role for nucleolin in post-stroke functional recovery.

## Resource availability

### Lead contact

Requests for further information and resources will be fulfilled by the lead contact, S. Thomas Carmichael (scarmichael@mednet.ucla.edu).

### Materials availability

Plasmids generated in this study are available upon request to the lead contact. Nucleolin heterozygous knockout mice are available from Jackson Laboratories (JAX 037120).

### Data and code availability

• Data: Data reported in this paper will be shared by the lead contact upon request.
• Code: Original code reported in this paper will be shared by the lead contact upon request.
• Additional resources: Any additional information required to reanalyze the data reported in this paper is available from the lead contact upon request.

## Acknowledgments

This work was supported by grants from the 10.13039/100005984Adelson Medical Research Foundation, the 10.13039/501100001742United States-Israel Binational Science Foundation (BSF grant 2023327), the UCLA-Weizmann Institute of Science Collaboration, and the Chaya Professorial Chair in Molecular Neuroscience (M.F.).

## Author contributions

Study design: S.P.B, M.F., and S.T.C. Study conduct: S.P.B., M.H., S.A., O.A., and I.R. Data analysis and interpretation: S.P.B., M.F., S.T.C. Supervision and funding acquisition: M.F. and S.T.C. Manuscript drafting and review: All authors.

## Declaration of interests

STC has received a research grant from Calico Laboratories and provided scientific consulting to Fibrobiologics, Inc.

## STAR★Methods

### Key resources table

**Table:** 

REAGENT or RESOURCE	SOURCE	IDENTIFIER
**Antibodies**		

Rabbit anti-flag	Invitrogen	PA1-984B RRID: AB_347227
Rat anti-GFAP	Invitrogen	13-0300 RRID:AB_2532994
Goat anti-Iba1	Abcam	ab5076 RRID:AB_2224402
Rabbit anti-nucleolin	Abcam	ab129200 RRID:AB_11144140
Guinea pig anti-NeuN	Synaptic Systems	266004 RRID:AB_2619988

**Bacterial and virus strains**		

pAAV-hsyn-cre-p2a-dendra2	This paper	N/A
pAAV-hsyn-cre-p2a-dendra2GARWT	This paper	N/A
pAAV-CAG-flex-Ruby2sm-Flag-WPRE	Addgene	98928-AAV1 RRID:Addgene_98928

**Critical commercial assays**		

HCR 3.0 Slc17a7	Molecular Instruments	N/A
HCR 3.0 Gad1	Molecular Instruments	N/A
HCR 3.0 Ncl	Molecular Instruments	N/A

**Experimental models: Organisms/strains**		

Nucleolin heterozygous knockout mice	Doron-Mandel et al.^15^, Jackson Laboratories	JAX 037120
C57Bl/6J	Jackson Laboratories	000664

**Software and algorithms**		

StereoInvestigator	MicroBrightfield	N/A
Neurolucida 360	MicroBrightfield	N/A
ImageJ	NIH	N/A
Graphpad Prism	Graphpad	N/A

### Experimental model details

All studies involving animals were performed in accordance with US National Institutes of Health Animal Protection Guidelines and the University of California Los Angeles Chancellor’s Animal Research Committee (ARC protocol 2000–159). All animals used were 8–12-week-old males at the time of stroke. Nucleolin heterozygous knockout and wild type littermates were described previously.^16^ C57Bl/6J mice were obtained from Jackson Laboratories (JAX ID #000664), and were randomly assigned to experimental groups prior to stroke. Mouse were housed 2–4 to a cage in 12:12 light:dark cycle, and unless otherwise noted had *ad lib* access to food and water.

### Method details

#### Photothrombotic stroke

Focal cortical ischemia was achieved using established protocols^6^^,^^28^ with some modifications. Briefly, mice were anesthetized with 4% isoflurane and placed in the stereotactic apparatus. The skull was exposed and dried, and bregma identified. A 525 nm laser, diameter 2mm, was targeted over the left forelimb motor cortex (A/P 0.1, M/L 1.5). Rose Bengal (10 mg/mL in saline) was injected (10 μL/g of body weight, typically 250 μL) intraperitoneally, and allowed to circulate for 5 min. The forelimb motor cortex was illuminated for 10 min at an intensity of 13 mW for axonal sprouting studies, or 18 mW for behavioral studies. Body temperature was maintained at 37C throughout surgery using a rectal probe. The scalp was closed using surgical adhesive, and the mouse returned to its home cage to recover.

#### Molecular biology and AAV generation

GARWT and Dendra2 AAVs were adapted from previous use.^16^ Briefly, cre-P2A was amplified from pAAV-hsyn-cre-p2a-dTomato, a gift from Rylan Larsen (Addgene #107738) and inserted immediately upstream of Dendra2 or GARWT through standard molecular protocols. AAVs were packaged via ultracentrifugation as described.^41^ Viruses were titered via qPCR, and diluted to a final concentration of 1.0x10^13^ GC/mL pAAV-CAG-Flex-Ruby2sm-Flag-WPRE-SV40 was a gift from Loren Looger (Addgene viral prep #98928-AAV1).

#### Virus/BDA injection

GARWT or Dendra2 viruses were injected into premotor cortex at the time of stroke. Immediately following stroke induction or sham stroke, a burr hole was drilled in the premotor cortex (A/P 1.5, M/L 1.75, D/V 0.75), and 200 nL of a 1:1 ratio of GAR targeting virus and Flex-smFLAG virus was injected at a rate of 1 nL/s using a nanoinjector (World Precision Instrument). In cases where BDA was injected, as an anterograde tracer, 300 nL was injected into premotor cortex 1 week prior to sacrifice as described above.

#### Grid-walk

The grid-walking task was performed as described previously.^27^^,^^28^^,^^30^ Briefly, 1 day prior to stroke or sham induction, and at designated time points after, mice were placed on an elevated grid and allowed to freely ambulate for 5 min, with footsteps recorded from below. An investigator blind to experimental condition evaluated these videos for a minimum of 2 min or 50 total steps, whichever came later. A step was defined as forward ambulation with all limbs. Foot faults were calculated as a proportion of total steps taken (number of foot faults/total steps taken).

#### Pasta matrix

The pasta matrix task was performed as described previously.^31^ Briefly, mice were individually placed in a clear container with a 1 cm opening to allow for grasping 3.3 cm pieces of pasta arranged in a 5x5 grid. Over 4 weeks of training, mice were assessed for forelimb dominance, and forced to use only that limb for grasping and breaking pieces of pasta. Following stroke, mice were assessed on a biweekly basis and allowed 15 min to break as many pieces of pasta possible. Results are reported as a percentage of baseline number of pasta pieces broken (number of broken pasta at time point/number of broken pasta at baseline). Mice that failed to break 5 pieces of pasta at baseline were excluded from this task post-stroke.

#### Flat map generation and quantification

Flattened sections were obtained as described previously.^6^^,^^12^^,^^28^ Briefly, mice were deeply anesthetized with isoflurane, transcardially perfused with ice-cold phosphate buffered saline and 4% paraformaldehyde, the cortex was isolated and flattened between two glass slides with 1 mm spacers, post-fixed for 24 h and cryoprotected in a 30% sucrose solution. Shelled cortices were sectioned on a cryostat tangentially, and a representative series encompassing all cortical layers were stained for FLAG epitope tag with appropriate secondary antibody. Flattened sections were imaged using StereoInvestigator (MBF) on a Nikon Eclipse Ni epifluorescent microscope. Axonal puncta were quantified as described previously using Neurolucida 360 (MBF), and spatial statistics were generated using a custom program.^29^

#### Immunohistochemistry and *in situ* hybridization

Mice were perfused and brains prepared as described earlier. Cortical sections were cut, and a cortical series encompassing anterior and posterior to the stroke were stained for GFAP and Iba1, with appropriate secondary antibodies. Sections were imaged at 20x on a Nikon Eclipse Ti. Infarct volume, ventricle volume and Iba1 intensity were measured using ImageJ.

For evaluation of nucleolin expression post-stroke, cortical sections were stained for nucleolin and NeuN 7-day post-stroke. *In situ* hybridization at various time points post-stroke was performed using custom Molecular HCR 3.0 probe sets against nucleolin, Slc17a7 and Gad1, in addition to DAPI following the manufacturer’s protocol. For *in situ* hybridization studies, tissue was imaged at 20x on a Zeiss LSM900 confocal microscope, masks were generated for Slc17a7^+^ or Gad1^+^ cell bodies, and puncta localized inside Slc17a7^+^ or Gad1^+^ masked cell bodies within 250 μm of the infarct border were counted using Imaris. Settings, such as intensity thresholding, were kept consistent for all groups. For protein localization studies, tissue was imaged at 63x on a Zeiss LSM900 confocal microscope, and nucelolin puncta within NeuN^+^ cell bodies within 250 μm of the infarct border were counted using ImageJ. All analyses were performed by an investigator blinded to either experimental group or time point post-stroke.

### Quantification and statistical analysis

#### Statistics

Spatial statistics were determined as described previously.^6^^,^^12^^,^^28^ Statistics for RNA or protein puncta counting, stroke volume and intensity measurements were either t-tests or 2-way ANOVA. Behavior statistics are 2-way repeated measure ANOVA (GraphPad Prism). Axonal puncta were organized into concentric circles, centered at the injection point (0,0), and a general linear mixed model with Tukey’s post-hoc correction was calculated using custom R script to determine significant differences between groups within concentric circles. Outliers were determined using the ROUT method with Q = 1%; one outlier was removed from all analyses. Sample sizes and statistical tests used are described in the figure legend and text of the paper.
